# Event and Comment

**Published:** 1920-02

**Authors:** 


### DENTAL REGISTER

THE MAGAZINE OF DENTISTRY

Vol. LXXIV FEBRUARY, 1920 No. 2

### EVENT AND COMMENT

**Passing of the Faithful** Our profession has been called upon with unusual frequency in the recent days to record the death of men who have devoted large portions of their time and talents to the building of our calling. Such occasions call for thoughtful marking on these events by appropriate reflections on the good qualities which endeared these men to us in life, and it should also make an enduring impression on our minds as to the benefits which such lives confer on the profession and humanity in general. It would require a careful and critical analysis of the excellencies which characterized the personalities and activities of the particular individuals to whom we have paid our parting respects in these last days, that we might appraise the value of their contributions in particulars or even in general estimates. Those who knew and worked with them can evaluate them in a measure, but always the personal element asserts itself in any attempt to estimate the worth of such lives to the cause of humanity. We instinctively cherish the memories of personal contacts that reacted to our own mental and sympathetic activities; but do we always credit the predominant motive which has kept such men faithful and constant in pursuing the ideals which have made their lives worth while? We have lost recently four men who will be seriously missed, because they gave things worth while to the particular vocation which absorbs our best endeavors. Drs. Henry W. Morgan, B. Holly Smith, W. W. Belcher, and Charles S. Butler were not brilliant men in the spectacular sense, but they were earnest-minded and devoted to the higher ideals. Each in his own way made a name and a reputation for himself, but each did a much better thing, because he gave of his resources unstintedly to making humanity better and happier. This is why we honor them. Is there any other way in which they could have done better for themselves?

**Dental Cosmos Celebrates a Birthday** The *Cosmos* issues a souvenir number with January, claiming its sixtieth anniversary. We do not quite understand how this is figured, as the *Cosmos* was first issued August, 1859; but this is a matter of slight consequence. August was a very hot month in which to hold a celebration, and besides it has taken time to prepare the historical material which is presented in this souvenir number. It is well done by master minds and is most refreshing as well as stimulating. We are glad to extend our cordial congratulations and also to commend the past achievements of this journal, which has most consistently represented the best in dentistry for all the years of its existence. It has not only had a long and honorable career of continuous publication, but it has faithfully recorded all the important developments of the science and art of dentistry during the period of its greatest development. The establishment of *The Journal of the National Dental Association* has taken from the *Cosmos* a large part of the literary output of the profession which formerly appeared in that journal, and this to some extent interrupts its representative career; but there is a world of new literature that must have a dignified medium of record and dissemination. If we can forecast the future of the *Cosmos* from its past traditions, we know now what will be its policy as an efficient and ethical representative of our profession's literary progress. With a great and resourceful organization back of it, which was founded and inspirited by a dentist who changed to a commercial career, that he might serve his profession more adequately, and who during his life steadfastly strove to furnish the profession with the best possible equipment for its artistic achievements, we feel confident of a still greater future for this representative organ of professional activities during the next sixty years. We also are reassured because the editorial management will continue in the hands of the present experienced and devoted editors, whom we know and unqualifiedly trust.

